# Inguinal single-port approach of endoscopic component separation for abdominal wall defects: A case series

**DOI:** 10.1016/j.amsu.2022.104611

**Published:** 2022-09-09

**Authors:** Mamoru Miyasaka, Yo Kawarada, Yoshiyuki Yamamura, Shuji Kitashiro, Shunichi Okushiba, Satoshi Hirano

**Affiliations:** a Department of Surgery, Tonan Hospital, Japan; b Department of Gastroenterological Surgery II, Hokkaido University Faculty of Medicine, Japan

**Keywords:** 1 endoscopic component separation, 2 single port, 3 inguinal incision, 4 abdominal wall defect, 5 case series, CS, component separation, ECS, endoscopic component separation

## Abstract

**Background:**

The component separation (CS) technique is widely used for abdominal wall defects, particularly in infected wounds. CS is associated with many wound complications due to subcutaneous blood flow disturbance. Endoscopic component separation (ECS) has fewer wound complications compared to CS and has been performed recently. However, there are various port required placements for ECS, and this technique requires proficiency. One approach for ECS is the inguinal single-port approach, which can be performed from an inguinal incision similar to that used in open surgery for inguinal hernias.

**Case presentation:**

We performed ECS with an inguinal single-port approach in three older adults. All patients had abdominal wall defects with infection at the central abdominal wound site. A 2–3-cm incision was created in the middle of the inguinal ligament, and a single-port surgical device with two 5-mm trocars was placed in the incision. The external oblique muscle was separated from the internal oblique muscle, and the external oblique aponeurosis was released. The muscle flap of the abdominal wall was moved to the central line. Tension-free abdominal wall closure was possible using a one-handed approach.

**Conclusions:**

ECS, which has fewer wound complications, requires proficiency. This procedure is a simple and easy-to-perform procedure using an inguinal incision that surgeons are familiar with.

## Highlights

• Component separation is associated with wound complications.
• Endoscopic component separation has fewer complications, but is not easy to approach.
• Inguinal single port approach for endoscopic component separation technique is easy.

## 1 Introduction

Ramirez et al. [1] first developed a component separation (CS) technique to close complicated abdominal wall defects. CS allows the abdominal wall to be closed without the use of artificial materials and is widely used as a safe and effective approach, particularly in previously infected fields. However, wide ligation of the abdominal wall perforators leads to wound complications [2]. Recently, a minimally invasive approach called the endoscopic component separation (ECS) technique has been reported to reduce wound complications by preserving perforating abdominal wall vessels [[2], [3], [4]]. Since ECS sometimes requires an endoscopic balloon insufflator and port settings vary depending on the reports, it is difficult to standardize the procedure [5,6]. Herein, we describe the inguinal single-port approach for ECS, which can be performed using an inguinal incision similar to that used in open surgery for inguinal hernias. This approach has the advantage of easy performance in areas surgeons are accustomed to.

## 2 Patient & method

We performed ECS by inguinal single-port approach for abdominal wall defects. Patients who had abdominal wall defects with infection at the central abdominal wound site were retrospectively enrolled at a single center (Tonan Hospital) between January 2020 and December 2021, excluding American Society of Anesthesiologists physical status classification (ASA-PS) ≥ 3. This study is registered with the ResearchRegistry and the unique identifying number is: researchregistry8254 (https://www.researchregistry.com/browse-the-registry#home/). This case series has been reported in line with the PROCESS Guideline [7]. All surgeries were performed by a team of experienced surgeons.

## 3 Technical approach

**Fig. 1:**
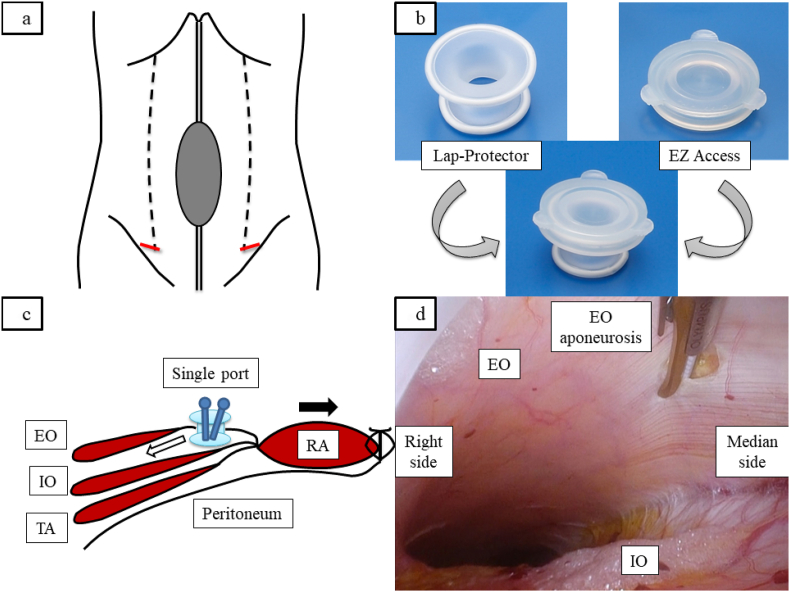
(a) Schema of the body surface. (b) Images of Lap-Protector and EZ Access and the two connections. (c) Schema of the abdominal wall. The area between the external and the internal oblique muscles is dissected. The myofascial flap can be mobilized medially. (d) The external oblique aponeurosis can be incised to the costal margin on the cranial side with manual compression from the skinEO, external oblique muscle; IO, internal oblique muscle; TA, transversus abdominis muscle; RA, rectus abdominis muscle.Red line, incision site in groin; black dotted line, outer edge of the rectus abdominis muscle; white arrow, dissection between the external and internal oblique muscles; black arrow, mobilization of the compound flap of the rectus abdominis and attached internal oblique/transverse abdominis muscle complex. . (For interpretation of the references to colour in this figure legend, the reader is referred to the Web version of this article.)

The midpoint of the inguinal ligament was marked from the pubis to the anterior superior iliac spine during surgery under general anesthesia. From the mark, a 2–3-cm linear incision parallel to the inguinal ligament similar to that used in classical open inguinal hernia repair. The surgeon dissected the subcutaneous fatty tissue until the external oblique muscle fascia was identified. After opening the external oblique fascia, a Lap-Protector (Hakko Co., Ltd., Nagano, Japan) with an outer diameter of 4 cm was inserted between the external oblique fascia and internal oblique muscle (Fig. 1a). An EZ Access (Hakko Co., Ltd., Nagano, Japan) equipped with two 5-mm trocars was set to inject CO_2_ gas at a pressure of 10 mmHg, and the external oblique muscle was separated from the internal oblique muscle by air pressure (Fig. 1b). The area between the external and internal oblique muscles was dissected easily, extending from the inguinal ligament to the level of the costal margin. All procedures up to this point were performed using only one hand. The external oblique aponeurosis was released from the external oblique muscle and its fascia through an incision 1–2 cm lateral margin at the site of fusion with the internal oblique muscle on the median side (Fig. 1c and d). The compound flap of the rectus abdominis and attached internal oblique/transversus abdominis muscle complex could now be mobilized medially. Finally, the surgeon closed the tension-released abdominal wall defect. Manual compression was useful for incision of the appropriate area.

## 4 Case reports

### 4.1 Case 1

**Fig. 2:**
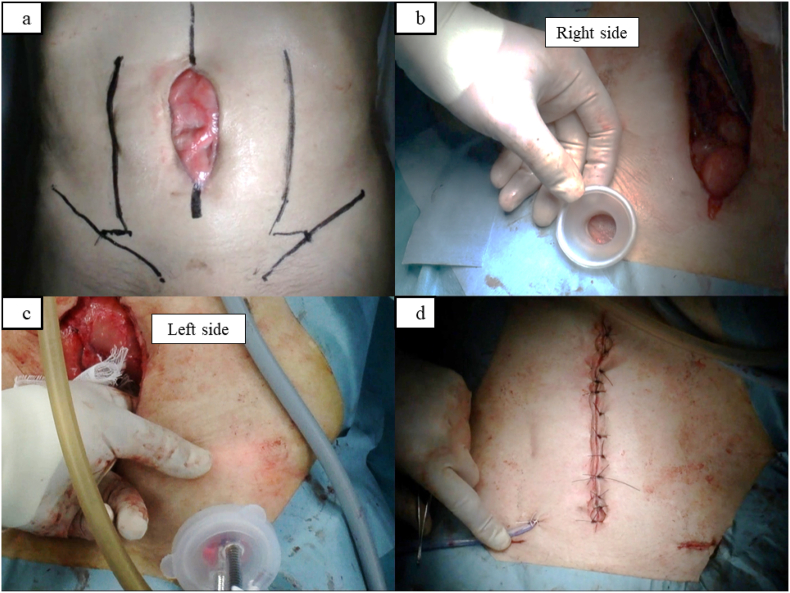
(a) Width of the abdominal wall defect was 6 cm, and the length was 13 cm. (b) The Lap-Protector is inserted into the inguinal incision. (c) Manual compression is performed on the skin surface. (d) The abdominal wall is closed with bilateral ECS.

An 80-year-old man who received small bowel resection because of strangulated intestinal obstruction developed abdominal midline wound dehiscence with infection. As delivered by Vacuum-Assisted Closure® (V.A.C.® Therapy, KCI Licensing, Inc San Antonio, TX), negative pressure wound therapy was used on the wound for approximately 2 weeks. However, the abdominal wall defect with exposed intestine and incisional hernias remained. The width of the defect was 6 cm, and the length was 13 cm (Fig. 2a). Surgery was performed to close the abdominal wall defect. As an operative procedure, the adhesion of the midline scar was dissected from the rectus abdominis muscle and peritoneum. The scar was placed in the abdominal cavity along with the intestines. Bilateral ECS by inguinal single-port approach was performed for abdominal closure (Fig. 2b–d). The total operative time of ECS was 20 min. The patient was discharged 3 weeks postoperatively.

### 4.2 Case 2

An 83-year-old woman who was treated for abdominal incisional hernias using an absorbable barrier mesh (25 × 15 cm, Ventrio™ ST Hernia Patch) suffered from an infection of the mesh and fistula of the small bowel and skin. After mesh removal and small bowel resection, bilateral ECS using the inguinal single-port approach was performed for abdominal closure. The operative time of ECS was 30 min. The patient was discharged 2 weeks postoperatively.

### 4.3 Case 3

An 80-year-old woman who had sigmoid colon perforation was received Hartmann's procedure [8]. Postoperatively, she had abdominal midline wound dehiscence with infection as in Case 1. Due to sigmoid colon stoma formation, unilateral (right side) ECS by an inguinal single-port approach was performed for abdominal closure. The operative time of ECS was 7 min. The patient was discharged 2 weeks postoperatively.

## 5 Discussion

The procedure for closing these complicated abdominal wall defects, the CS technique was developed by Ramirez et al. [1] in 1990. CS is a technique that allows re-establishment of a functional abdominal wall with autologous tissue repair. The procedure releases the rectus abdominis muscle from its posterior rectus sheath by dividing the external oblique aponeurosis and separating the space between the external and internal oblique muscles. The muscle flap consisting of the rectus, internal oblique, and transverse abdominis can be mobilized medially. Allowing movement of approximately 10 cm on each side, tension-free midline fascial closure is achieved [1]. This technique is now commonly called “anterior” CS because a transversus abdominis release technique is available [9,10].

Classical open CS may cause wound complications [2,3]. Creating large subcutaneous skin flaps requires ligation of perforating abdominal wall blood vessels. Decreased blood flow to the skin causes many complications, such as ischemia, infection, and hematomas [2.3]. Wound infection rates are approximately 20%–50% [[11], [12], [13], [14]].

ECS was developed to reduce invasiveness and wound complications by protecting perforating abdominal wall vessels [2,3]. In addition to open CS, bilateral incisions of the external oblique aponeurosis were made, and the avascular area between the external oblique and internal oblique muscles was separated using an endoscope. While the distance that the myofascial flap can be mobilized to the midline is 86% shorter in ECS than in open CS, ECS can preserve the tissue blood supply, improve resistance to infection, and promote tissue healing [[15], [16], [17]]. Some meta-analyses have shown that ECS has fewer wound complications than open CS [4,18]. It is possible to relatively easily expand the separation area from the inguinal ligament to the level of the costal margin in the ECS. However, inserting the trocar between the external oblique and internal oblique muscles is difficult, and many ECS procedures require a balloon dissector and a 10–12-mm port and one to two additional 5-mm ports [2,3]. There are also reports on procedures using Yankauer suction and handheld electrocautery and on a single-port approach as in our cases; thus, it remains difficult to standardize the procedure [12,19].

The advantage of our approach is that surgeons can use the same approach as open inguinal hernia repair. Even with the single-port approach, the external oblique muscle aponeurosis can be easily incised using only one hand by deforming the abdominal wall with manual compression from the skin surface. It does not require time to get accustomed to, and the endoscope is only used for approximately 10–20 min on each side. Because the number of cases is still small, we should be able to save even more time in the future. For wound dehiscence after Hartmann's procedure, as in our case, abdominal wall closure can be easily performed using unilateral ECS. As it does not require an endoscopic balloon insufflator, and therefore, this method may also reduce costs.

This report had several limitations. The number of cases was small. The main purpose of the surgery is to close the abdominal wall to protect the internal organs, and the procedure may be insufficient as a radical treatment for ventral hernia. However, it is considered a simple approach to abdominal wall defects depending on the case, such as in bedridden older patients. Patients 1 and 3 were also inactive, bedridden patients, and treatment of the ventral hernia was not the primary goal of the surgery.

To the best of our knowledge, there are few reports on the inguinal single-port approach of ECS for abdominal wall defects in the English literature. ECS is a minimally invasive surgery with fewer wound complications for the repair of abdominal wall defects. The difficulty of this approach may be resolved using our method. Further studies are required to generalize this procedure.

## 6 Conclusions

ECS is a minimally invasive procedure that has been widely performed recently, with fewer wound complications. To the best of our knowledge, this is one of the few reports on the inguinal single-port approach of ECS for abdominal wall defects. This approach may help overcome the difficulty of this approach in ECS.

## Ethical approval

This is an observational study. The Tonan Hospital Research Ethics Committee has confirmed that no ethical approval is required.

## Please state any sources of funding for your research

This research did not receive any specific grant from funding agencies in the public, commercial, or not-for-profit sectors.

## Author contribution

Conception and design of study; M. Miyasaka, Y. Kawarada. Acquisition of data; M. Miyasaka, Y. Kawarada, Y. Yamamura, S. Kitashiro, S. Okushiba. Analysis and/or interpretation of data: M. Miyasaka, Y. Kawarada. Drafting the manuscript: M. Miyasaka, Y. Kawarada. Revising the manuscript critically for important intellectual content: Y. Kawarada, S. Hirano. Approval of the version of the manuscript to be published (the names of all authors must be listed): M. Miyasaka, Y. Kawarada, Y. Yamamura, S. Kitashiro, S. Okushiba, S. Hirano.

## Please state any conflicts of interest

There are no conflicts of interest to declare.

## Registration of research studies

This study is registered with the ResearchRegistry and the unique identifying number is: researchregistry8254 (https://www.researchregistry.com/browse-the-registry#home/).

## Guarantor

Mamoru Miyasaka, Yo Kawarada.

## Consent

The subjects provided informed consent, and patient anonymity was preserved.

## Provenance and peer review

Not commissioned, externally peer-reviewed.

## References

[1] Ramirez O.M., Ruas E., Dellon A.L. (1990). “Components separation” method for closure of abdominal wall defects: an anatomic and clinical study. Plast. Reconstr. Surg..

[2] Maas S.M., de Vries R.S., van Goor H. (2002). D. de Jong, R. P. Bleichrodt. Endoscopically assisted “components separation technique” for the repair of complicated ventral hernias. J. Am. Coll. Surg..

[3] Lowe J.B., Garza J.R., Bowman J.L., Rohrich R.J., Strodel W.E. (2000). Endoscopically assisted “components separation” for closure of abdominal wall defects. Plast. Reconstr. Surg..

[4] Switzer N.J., Dykstra M.A., Gill R.S., Lim S., Lester E., de Gara C. (2015). Endoscopic versus open component separation: systematic review and meta-analysis. Surg. Endosc..

[5] Mommers E.H.H., Wegdam J.A., Nienhuijs S.W., de Vries Reilingh T.S. (2016). How to perform the endoscopically assisted components separation technique (ECST) for large ventral hernia repair. Hernia.

[6] Earle D. (2016).

[7] Agha R.A., Sohrabi C., Mathew G., Franchi T., Kerwan A., O'Neill N for the PROCESS Group (2020). The PROCESS 2020 guideline: updating Consensus preferred reporting of CasE series in surgery (PROCESS) Guidelines. Int. J. Surg..

[8] Hartmann H. (1921).

[9] Krpata D.M., Blatnik J.A., Novitsky Y.W., Rosen M.J. (2012). Posterior and open anterior components separations: a comparative analysis. Am. J. Surg..

[10] Novitsky Y.W., Elliott H.L., Orenstein S.B., Rosen M.J. (2012). Transversus abdominis muscle release: a novel approach to posterior component separation during complex abdominal wall reconstruction. Am. J. Surg..

[11] Albright E., Diaz D., Davenport D., Roth J.S. (2011). The component separation technique for hernia repair: a comparison of open and endoscopic techniques. Am. Surg..

[12] Ghali S., Turza K.C., Baumann D.P., Butler C.E. (2012). Minimally invasive component separation results in fewer wound-healing complications than open component separation for large ventral hernia repairs. J. Am. Coll. Surg..

[13] Clarke J.M. (2010). Incisional hernia repair by fascial component separation: results in 128 cases and evolution of technique. Am. J. Surg..

[14] Harth K.C., Rosen M.J. (2010). Endoscopic versus open component separation in complex abdominal wall reconstruction. Am. J. Surg..

[15] Bachman S.L., Ramaswamy A., Ramshaw B.J. (2009). Early results of midline hernia repair using a minimally invasive component separation technique. Am. Surg..

[16] Saulis A.S., Dumanian G.A. (2002). Periumbilical rectus abdominis perforator preservation significantly reduces superficial wound complications in ‘‘separation of parts’’ hernia repairs. Plast. Reconstr. Surg..

[17] Rosen M.J., Williams C., Jin J., McGee M.F., Schomisch S., Marks J. (2007). Laparoscopic versus open-component separation: a comparative analysis in a porcine model. Am. J. Surg..

[18] Jensen K.K., Henriksen N.A., Jorgensen L.N. (2014). Endoscopic component separation for ventral hernia causes fewer wound complications compared to open components separation: a systematic review and meta-analysis. Surg. Endosc..

[19] Elstner K.E., Read J.W., Jacombs A.S.W., Martins R.T., Arduini F., Cosman P.H. (2018). Single port component separation: endoscopic external oblique release for complex ventral hernia repair. Surg. Endosc..

